# Implications of Updating the Minimum Dietary Diversity for Children Indicator for Tracking Progress in the Eastern and Southern Africa Region

**DOI:** 10.1093/cdn/nzaa141

**Published:** 2020-08-21

**Authors:** Rebecca A Heidkamp, Yunhee Kang, Kudakwashe Chimanya, Aashima Garg, Joan Matji, Mara Nyawo, Hope Craig, Mary Arimond, Andrew L Thorne Lyman

**Affiliations:** Department of International Health, Johns Hopkins Bloomberg School of Public Health, Baltimore, MD, USA; Department of International Health, Johns Hopkins Bloomberg School of Public Health, Baltimore, MD, USA; UNICEF Eastern and Southern Africa Regional Office, Nairobi, Kenya; UNICEF Program Division, New York, NY, USA; UNICEF Eastern and Southern Africa Regional Office, Nairobi, Kenya; UNICEF Eastern and Southern Africa Regional Office, Nairobi, Kenya; Department of International Health, Johns Hopkins Bloomberg School of Public Health, Baltimore, MD, USA; Independent Consultant, Takoma Park, MD, USA; Department of International Health, Johns Hopkins Bloomberg School of Public Health, Baltimore, MD, USA

**Keywords:** children under 5, dietary diversity, breastfeeding, program monitoring, infant, child, nutrition, animal source food, vegetable, fruit

## Abstract

Minimum dietary diversity (MDD), a population-level dietary quality indicator, is commonly used across low- and middle-income countries to characterize diets of children aged 6–23 mo. The WHO and UNICEF recently updated the MDD definition from consumption of ≥4 of 7 food groups in the previous 24 h (MDD-7) to ≥5 of 8 food groups (MDD-8), adding a breastmilk group. The implications of this definition change were examined across 14 countries in Eastern and Southern Africa where improving complementary feeding is a policy priority. A lower MDD-8 score was found compared with MDD-7 across all countries; in 3 countries the difference between indicators was >5 percentage points. Country-level variability is driven by differences in breastfeeding rates and dietary diversity score. As countries transition to the new indicator it is important to actively publicize changes and to promote valid interpretation of MDD trends.

## Teaser

We examine the implications of recent changes in indicator definition for national estimates of dietary quality among children aged 6–23 mo in countries where improving child feeding is a policy priority.

## Introduction

Poor quality diet during early childhood is a risk factor for multiple forms of malnutrition and related health outcomes including undernutrition, micronutrient deficiencies, as well as obesity and nutrition-related noncommunicable disease (1–3). The WHO-recommended Essential Nutrition Actions include the promotion of appropriate complementary feeding practices in children aged 6–23 mo. Key practices include the gradual introduction of a diverse variety of age-appropriate foods with continued breastfeeding (4). In 2018, 116 UNICEF-supported countries globally reported the existence of programs to improve complementary feeding (5). In 2019, 19 of 21 countries in the UNICEF Eastern and Southern Africa (ESA) region had a specific programmatic focus on improving dietary diversity in this age group.

Because improving the quality of young children's diets is a priority, the regular collection of data that can be used to measure the impact of programs and policies on children's diets is important. In 2008 WHO and UNICEF released a set of global standard indicators for infant and young child feeding (IYCF) practices that includes minimum dietary diversity (MDD), a qualitative dietary quality indicator specific to children aged 6–23 mo (6). Compared with more resource-intensive quantitative measures of food intake based on 24-h recalls or FFQs, dietary diversity scores are simple indicators that reflect the number of individual food items or food groups consumed over a period of time (7–9). The MDD score for children aged 6–23 mo reflects food groups consumed in the previous 24 h and has been validated against the micronutrient density of infant diets in some settings (10–12). It is collected as part of large-scale, multitopic surveys such as the Demographic and Health Surveys (DHS).

MDD for children aged 6–23 mo is commonly included in national nutrition monitoring frameworks across low- and middle-income countries. For example, in the ESA region, a number of countries including Ethiopia, Kenya, Malawi, and Zimbabwe included MDD as a key indicator in targets or documents. MDD and the related indicator minimum acceptable diet (MAD) are also prioritized by donors such as the US Agency for International Development (13) and tracked by global accountability initiatives including the Global Nutrition Report (14).

The earlier MDD indicator definition, which we refer to as MDD-7, was based on consumption of ≥4 of 7 food groups including a combined group for dairy and infant formula or other breastmilk substitutes; it did not account for breastmilk consumption because the rationale was to isolate the “complementary feeding diet” (15). Separate indicators were reported for breastfeeding practices. This guidance cautioned against combining or comparing MDD-7 across breastfed and nonbreastfed children in the same population because nonbreastfed children who receive milk and/or infant formula can have higher scores than breastfed children who do not receive these items. In 2017, the WHO-UNICEF Technical Expert Advisory Group for Nutrition Monitoring released an updated MDD definition as part of the Global Nutrition Monitoring Framework Monitoring and Evaluation guidance, which we refer to as MDD-8, that includes breastmilk as an eighth food group and increased the threshold to ≥5 of 8 food groups (16). MDD-8 can be compared across breastfed and nonbreastfed groups. In 2019, UNICEF updated the MDD definition from MDD-7 to MDD-8 in its global databases (17). Updated IYCF indicator guidance is expected from WHO and UNICEF in 2020. The DHS has adopted MDD-8, starting in 2020 as part of Round 8 survey reporting.

The implications of shifting from MDD-7 to MDD-8 for tracking country-level progress have not been well studied. With this in mind, the aim of this article is to compare estimates for MDD-7 and MDD-8 using data from 14 countries in UNICEF ESA region, to identify common reasons for divergence in the 2 indicators, and to reflect on the implications for interpretation and use by national actors.

## Methods

For our analysis we used data from the most recent publicly available DHS datasets for 14 countries in the UNICEF ESA region: Angola 2015–16, Burundi 2016–17, Ethiopia 2016, Kenya 2014, Lesotho 2014, Malawi 2015–16, Mozambique 2011, Congo DRC 2013–14, Rwanda 2014–15, South Africa 2016, Tanzania 2015–16, Uganda 2016, Zambia 2013–14, and Zimbabwe 2015. The DHS surveys are implemented by national institutions in collaboration with ICF International (18).

In the DHS, questions about dietary intake are asked to caretakers of the youngest 6–23-mo-old child in each household. MDD-7 is defined as the proportion of children aged 6–23 mo who consumed ≥4 of 7 food groups in the previous 24 h. Food groups include: *1*) grains, roots, and tubers; *2*) legumes and nuts; *3*) dairy products; *4*) flesh foods (meat, fish, poultry, liver/organ meats); *5*) eggs; *6*) provitamin A–rich fruits and vegetables; and *7*) other fruits and vegetables (6). MDD-8 is defined as the proportion of children aged 6–23 mo who consumed ≥5 of 8 food groups in the previous 24 h. Food groups include the 7 listed above and an additional breastmilk group (16). We also calculated the mean number of food groups consumed by children aged 6–23 mo for each country using both the 7 and 8 food groups as well as the proportions of children aged 6–11 mo and 12–23 mo that consumed breastmilk in the previous day. The arithmetic difference between proportions of MDD-7 and MDD-8 was calculated for each country. Lowess curves (bandwidth 0.7) were generated to present the change across the 6–23-mo age range in the proportion of MDD-7, MDD-8, and currently breastfed. Except the Lowess analyses, DHS sample weights that account for the cluster survey design were used to generate nationally representative point estimates with 95% CI for prevalence and SE for sample means. All analyses were done in Stata version IC14 (StataCorp LLC).

## Results

**FIGURE 1:**
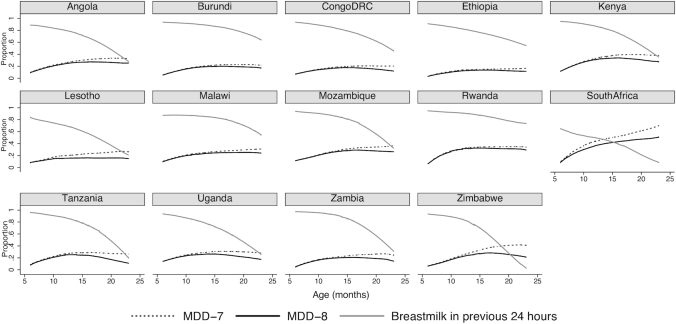
Prevalence of infant and young child feeding practices (breastmilk in previous 24 h, MDD-7, MDD-8) by age in months in children aged 6–23 mo in 14 countries in UNICEF Eastern and Southern Africa region. MDD, minimum dietary diversity; MDD-7, indicator definition for consumption of ≥4 of 7 food groups in the previous 24 h; MDD-8, indicator definition for consumption of ≥4 of 8 food groups in the previous 24 h. Data from reference 18.

As expected by definition, point estimates for MDD-8 (including breastmilk) in children aged 6–23 mo were consistently lower than MDD-7 (no breastmilk) across all countries; however, the magnitude of the difference varied. The Republic of South Africa had the largest difference (8.9 percentage points) and Ethiopia the smallest (1.4 percentage points). CIs around the point estimates for MDD-7 and MDD-8 overlapped in most countries, but did not overlap in South Africa, Tanzania, Uganda, Tanzania, and Zimbabwe (**Table 1**). The gap between MDD-7 and MDD-8 widened by child age, and in most countries, differences did not become apparent until the second year of life (**Figure 1**).

**TABLE 1:** Comparison of breastmilk consumption and dietary diversity scores in children aged 6–23 mo from 14 countries in the UNICEF Eastern and Southern Africa region^1^

					Dietary diversity (6–23 mo)				
			Breastmilk in previous 24 h		7 food groups (MDD-7)		8 food groups (MDD-8)		Difference (MDD-8 − MDD-7)
		*n* (6–23-mo-olds)	6–11 mo	12–23 mo	MDD ≥4	No. of groups consumed	MDD ≥5	No. of groups consumed	
Country	Survey year		% (95% CI)	% (95% CI)	% (95% CI)	Mean (SE)	% (95% CI)	Mean (SE)	% points
Angola	2015–16	4259	91.8 (89.5, 93.6)	63.7 (58.9, 64.2)	31.5 (28.7, 34.5)	2.7 (0.06)	27.8 (25.2, 30.6)	3.5 (0.06)	−3.7
Burundi	2016–17	4054	96.4 (94.9, 97.5)	85.3 (83.7, 86.7)	18.4 (17.0, 20.0)	2.4 (0.03)	16.6 (15.1, 18.1)	3.3 (0.03)	−1.8
Congo-DRC	2013–14	5221	96.0 (94.2, 97.3)	75.4 (73.1, 77.5)	19.4 (16.7, 22.1)	2.3 (0.05)	16.5 (14.2, 19.1)	3.1 (0.05)	−2.9
Ethiopia	2016	2965	93.4 (89.3, 96.1)	79.3 (76.3, 82.1)	13.4 (11.1, 15.7)	1.8 (0.05)	12.0 (9.8, 14.2)	2.7 (0.06)	−1.4
Kenya	2014	2906	97.3 (95.3, 98.5)	70.9 (68.0, 73.6)	40.1 (37.3, 42.9)	3.1 (0.05)	35.4 (32.7, 38.1)	3.9 (0.05)	−4.7
Lesotho	2014	957	83.9 (78.2, 88.3)	52.7 (48.1, 57.2)	23.2 (19.7, 26.8)	2.5 (0.01)	17.0 (14.1, 20.0)	3.2 (0.01)	−6.2
Malawi	2015–16	4879	91.9 (89.9, 93.5)	80.5 (78.6, 82.2)	24.7 (22.8, 26.5)	2.5 (0.03)	22.2 (20.5, 24.0)	3.3 (0.03)	−2.5
Mozambique	2011	3339	93.5 (91.6, 95.0)	73.3 (70.9, 75.6)	29.9 (28.0, 32.3)	2.8 (0.05)	27.4 (25.7, 30.0)	3.6 (0.05)	−2.5
Rwanda	2014–15	2430	97.0 (95.5, 98.0)	86.7 (84.7, 88.5)	29.5 (26.7, 32.2)	2.7 (0.04)	27.4 (24.3, 29.9)	3.6 (0.04)	−2.1
South Africa	2016	872	61.1 (52.1, 69.3)	34.7 (30.0, 39.6)	47.4 (43.4, 51.2)	3.5 (0.08)	38.5 (35.0, 42.1)	4.0 (0.07)	−8.9
Tanzania	2015–16	3170	96.5 (94.6, 97.7)	68.2 (65.8, 70.5)	25.6 (23.4, 27.9)	2.7 (0.04)	20.7 (18.7, 22.7)	3.5 (0.04)	−4.9
Uganda	2016	4418	94.8 (93.2, 96.0)	61.3 (59.1, 63.5)	28.6 (26.7, 30.5)	2.7 (0.04)	24.0 (22.2, 25.7)	3.5 (0.04)	−4.6
Zambia	2013–14	3776	96.6 (95.1, 97.7)	72.5 (70.2, 74.7)	21.8 (19.8, 23.7)	2.5 (0.03)	18.2 (16.4, 19.9)	3.3 (0.03)	−3.6
Zimbabwe	2015	1656	96.1 (93.8, 97.6)	57.0 (53.6, 60.4)	28.2 (25.6, 30.9)	2.7 (0.05)	21.5 (19.0, 23.9)	3.4 (0.05)	−6.7

1 MDD, minimum dietary diversity; MDD-7, 7-item minimum dietary diversity score; MDD-8, revised 8-item dietary diversity score. Data from reference 18.

Variability in breastfeeding rates in the second year of life appeared to be a major factor driving the differences in MDD-8 and MDD-7 across countries (Table 1, Figure 1). Consumption of breastmilk in the previous 24 h by 6–11-mo-olds was high (>88%) in most countries, but slightly lower in Lesotho (83.9%) and much lower in South Africa (61.1%). Breastfeeding rates declined with age across all countries (Table 1, Figure 1) but the prevalence of continued breastfeeding among 12–23-mo-olds varied from a high of 86.7% in Rwanda to a low of 34.7% in South Africa (Table 1).

The mean number of food groups consumed in all countries fell below the respective ≥5 or ≥4 cutoff of MDD-8 and MDD-7. In Ethiopia, the country with the lowest average number of food groups, the low mean meant that there was little difference in estimates of MDD-7 and MDD-8. In contrast, South Africa had the highest mean number of food groups and highest MDD rates regardless of whether 7 or 8 food groups were considered in the calculation. However, the rates of breastfeeding in South Africa were much lower, which contributed to it being the country with the largest gap between MDD-7 and MDD-8.

## Discussion

The shift from MDD-7 to MDD-8 involved 2 changes to the dietary diversity score: the addition of breastmilk as a group and the shifting of the cutoff from 4 to 5 groups. These changes had variable effects on the prevalence of MDD across countries in the ESA region. In most countries the proportion of children meeting MDD-7 compared with MDD-8 differed by <5 percentage points; however, in certain countries, particularly those with lower rates of breastfeeding in the second year of life, the divergence was more marked.

This shift has implications for how the indicator should be interpreted and used. Until now, the generally accepted pattern was that dietary diversity increases with age across the complementary feeding period. We see in Figure 1 that this holds for MDD-7, which increases and levels off over time; however, MDD-8 decreases after 18 mo in several countries, likely reflecting the cessation of breastfeeding. Breastmilk is not a complementary food, which was the rationale for its exclusion from the MDD-7 definition. However, continued breastfeeding during the complementary feeding period is recommended because breastmilk is also an important source of nutrients for the breastfed child (19). The MDD-7 indicator left out an important component of the diet, and calculation was also difficult due to the need to separately calculate the indicator for breastfed and nonbreastfed children. The MDD-8 indicator can be viewed as a more comprehensive picture of overall diet quality in children aged 6–23 mo and could lead to the identification of dietary deficiencies that were not so apparent when using MDD-7. For example, our analyses suggest a need to strengthen promotion of continued breastfeeding through the second year of life across the ESA region.

It is important to consider the significance of changing the MDD definition from the perspective of different stakeholders who use the indicator. As mentioned in the Introduction, the MDD-8 definition has already been taken up by some key global data and accountability stakeholders. Estimates based on the revised definition are available for all survey years captured in the publicly available UNICEF IYCF database (20). These values have been published with explanatory text in reports from UNICEF and other accountability initiatives including the Global Nutrition Report.

Even though key global stakeholders have adopted MDD-8, there is little evidence to suggest it has been taken up by country-level actors in the region. The revised IYCF indicator guidance document has not yet been published, and there has been limited communication to country-level policymakers and program implementer audiences. It is less common for country-focused actors to consult global databases, relying instead on survey reports and administrative systems (21). There is need for clear guidance and tools to strengthen country-led reporting of data (22).

The transition from MDD-7 to MDD-8 will have pragmatic implications for country-level progress tracking because many countries and programs have set and publicized targets based on the MDD-7 definition. The minimum acceptable diet (MAD) indicator, which relies in part on the MDD-7 indicator, is also used in nutrition strategies and plans in the ESA region. Most countries rely on point estimates alone for establishing baselines and monitoring progress—which means even a change that is not statistically significant could be interpreted as meaningful. For example, in 3 countries from the region with data from consecutive DHS surveys conducted about 5 y apart (Ethiopia, Rwanda, Malawi), the absolute annual change in MDD-7 averaged between 0.8 and 1.8 percentage points per year (23–28). Given how slowly the national estimates for the MDD indicator change in many of these countries, a change in indicator definition from MDD-7 to MDD-8 that brings a point estimate down by as little as 1.4 percentage points, as we saw in Ethiopia, is still a meaningful “regression” in progress if it is not interpreted correctly. Effort will be needed to disseminate information about how the indicator should be interpreted at the country level and to support the adjustment of MDD and MAD targets because the calculation of trends should not include a mix of MDD-7 and MDD-8 data points.

The addition of continued breastfeeding could also spur the need to consider how to accelerate progress in addressing continued breastfeeding after 12 mo, particularly for urban populations. This action could include policies such as flexible working arrangements and breastfeeding-friendly workplaces.

The nutrition community faced a similar transition with the introduction of the WHO 2006 Growth Standard in place of the National Center for Health Statistics reference for calculating anthropometric indicators. It took several years for countries to consistently use the revised standards and estimates (29). It is important to learn from this experience and to accompany the new MDD indicator guidance with outreach and technical support using simple communication tools to ensure buy-in.

The forthcoming release of updated WHO-UNICEF IYCF indicator guidance is an important opportunity to publicize the updated definition, to ensure the MDD-8 indicator is understood and consistently adopted, and to more generally reinforce the critical importance of diet quality in young children.

Future research priorities include validating MDD-8 against nutrient adequacy in different settings throughout the world, an effort that is already underway, as well as exploring the comparative strength of association of MDD-7 compared with MDD-8 for measures of growth, health, and other parameters of well-being.

## Notes

Funding for this research report was provided by the Bill & Melinda Gates Foundation.

Author disclosures: The authors report no conflicts of interest.

Abbreviations used: DHS, Demographic and Health Surveys; ESA, UNICEF Eastern and Southern Africa; IYCF, infant and young child feeding; MAD, minimum acceptable diet; MDD, minimum dietary diversity; MDD-7, indicator definition for consumption of ≥4 of 7 food groups in the previous 24 h; MDD-8, indicator definition for consumption of ≥4 of 8 food groups in the previous 24 h.

## References

[1] Afshin A, Sur PJ, Fay KA, Cornaby L, Ferrara G, Salama JS, Mullany EC, Abate KH, Abbafati C, Abebe Z Health effects of dietary risks in 195 countries, 1990–2017: a systematic analysis for the Global Burden of Disease Study 2017. Lancet. 2019;393(10184):1958–72. 30954305 10.1016/S0140-6736(19)30041-8 PMC6899507

[2] Bailey RL, West KP Jr, Black RE The epidemiology of global micronutrient deficiencies. Ann Nutr Metab. 2015;66(Suppl 2):22–33. 10.1159/000371618 26045325

[3] GBD Risk Factors Collaborators Global, regional, and national comparative risk assessment of 79 behavioural, environmental and occupational, and metabolic risks or clusters of risks in 188 countries, 1990–2013: a systematic analysis for the Global Burden of Disease Study 2013. Lancet. 2015;386(10010):2287. 26364544 10.1016/S0140-6736(15)00128-2 PMC4685753

[4] World Health Organization Complementary feeding: report of the global consultation, and summary of guiding principles for complementary feeding of the breastfed child. [Internet] WHO; 2003; [cited May 9, 2020]. Available from: https://apps.who.int/iris/handle/10665/42739.

[5] United Nations Children's Fund UNICEF Nutridash 2.0. [Internet] UNICEF; 2020; [cited May 9, 2020]. Available from: https://www.unicefnutridash.org/surveyreport/2.

[6] World Health Organization Indicators for assessing infant and young child feeding practices: part 1: definitions: conclusions of a consensus meeting held 6–8 November 2007in Washington DC, USA [Internet] WHO; 2008; [cited May 9, 2020]. Available from: https://apps.who.int/iris/bitstream/handle/10665/43895/9789241596664_eng.pdf?sequence = 1.

[7] Gibson RS, Charrondiere UR, Bell W Measurement errors in dietary assessment using self-reported 24-hour recalls in low-income countries and strategies for their prevention. Adv Nutr. 2017;8(6):980–91. 29141979 10.3945/an.117.016980 PMC5683000

[8] Ruel MT Is dietary diversity an indicator of food security or dietary quality? A review of measurement issues and research needs. [Internet] Food Consumption and Nutrition Division, International Food Policy Research Institute; 2003; [cited May 9, 2020]. Available from: http://ebrary.ifpri.org/utils/getfile/collection/p15738coll2/id/66936/filename/66937.pdf. 10.1177/156482650302400210 12891828

[9] Willett W Nutritional epidemiology. Vol. 40. Oxford University Press; 2012.

[10] Moursi MM, Arimond M, Dewey KG, Treche S, Ruel MT, Delpeuch F Dietary diversity is a good predictor of the micronutrient density of the diet of 6- to 23-month-old children in Madagascar. J Nutr. 2008;138(12):2448–53. 19022971 10.3945/jn.108.093971

[11] Working Group on Infant and Young Child Feeding Indicators Developing and validating simple indicators of dietary quality and energy intake of infants and young children in developing countries: summary of findings from analysis of 10 data sets. [Internet] Food and Nutrition Technical Assistance (FANTA) Project, Academy for Educational Development (AED); 2006; [cited May 26, 2020]. Available from: https://www.fantaproject.org/sites/default/files/resources/IYCF_Datasets_Summary_2006.pdf.

[12] Working Group on Infant and Young Child Feeding Indicators Developing and validating simple indicators of dietary quality of infants and young children in developing countries: additional analysis of 10 data sets. [Internet] Food and Nutrition Technical Assistance (FANTA) Project, Academy for Educational Development (AED); 2007; [cited May 26, 2020]. Available from https://www.fantaproject.org/sites/default/files/resources/IYCF_Datasets_2007.pdf

[13] United States Agency for International Development Feed the Future Handbook of Indicator Definitions. September 2019. [Internet] Washington (DC): USAID; 2019 Available from: https://www.agrilinks.org/sites/default/files/ftf-indicator-handbook-march-2018-508.pdf.

[14] Haddad L, Achadi E, Bendech MA, Ahuja A, Bhatia K, Bhutta Z, Blössner M, Borghi E, Colecraft E, De Onis M The Global Nutrition Report 2014: actions and accountability to accelerate the world's progress on nutrition. J Nutr. 2015;145(4):663–71. 25740908 10.3945/jn.114.206078 PMC5129664

[15] World Health Organization Indicators for assessing infant and young child feeding practices: part 2: measurement. WHO; 2010; [cited May 9, 2020] [Internet]. Available from: https://apps.who.int/iris/bitstream/handle/10665/44306/9789241599290_eng.pdf?ua =.

[16] World Health Organization, United Nations Children's Fund Global nutrition monitoring framework: operational guidance for tracking progress in meeting targets for 2025. [Internet] WHO; December 2017; [cited May 9, 2020]. Available from: https://www.who.int/publications-detail/9789241513609.

[17] United Nations Children's Fund UNICEF global IYCF database. [Internet] UNICEF; 2020; [cited May 9, 2020]. Available from: http://data.unicef.org/topic/nutrition/infant-and-young-childfeeding.

[18] ICF International The DHS program: data. [Internet] Rockville (MD): ICF; 2020; [cited August 3, 2020]. Available from: https://dhsprogram.com/data/.

[19] Pan American Health Organization, World Health Organization Guiding principles for complementary feeding of the breastfed child. [Internet] PAHO, WHO; 2003; [cited May 9, 2020]. Available from: https://iris.paho.org/handle/10665.2/752.

[20] United Nations Children's Fund UNICEF infant and young child feeding. [Internet] UNICEF; 2019; [cited May 9, 2020]. Available from: https://data.unicef.org/topic/nutrition/infant-and-young-child-feeding/.

[21] Buckland A, Aung T, King S, Manorat R, Becker L, Piwoz E, Rawat R, Heidkamp R, Thorne-Lyman A Nutrition data use and needs: findings from an online survey of global nutrition stakeholders. Curr Dev Nutr. [Internet] 2019;3(Suppl 1). Available from: 10.1093/cdn/nzz042.P22-003-19. PMC7688248 33282221

[22] Piwoz E, Rawat R, Fracassi P, Kim D Strengthening the nutrition data value chain for accountability and action: progress, gaps and next steps. Sight Life Mag. [Internet] 2019:33(1). Available from: https://www.anh-academy.org/sites/default/files/SightandLifeMagazine_2019_Data_in_Nutrition_StrengtheningtheDataValueChain-AccountabilityandAction.pdf.

[23] National Statistical Office/Malawi, ICF Malawi Demographic and Health Survey 2015–16. [Internet] Zomba, Malawi: National Statistical Office and ICF; 2017; [cited August 3, 2020]. Available from: http://dhsprogram.com/pubs/pdf/FR319/FR319.pdf.

[24] National Statistical Office (NSO)/Malawi, ICF Macro Malawi Demographic and Health Survey 2010. [Internet] Zomba, Malawi: NSO/Malawi and ICF Macro; 2011; [cited August 3, 2020]. Available from: http://dhsprogram.com/pubs/pdf/FR247/FR247.pdf.

[25] National Institute of Statistics of Rwanda, Finance Mo, Economic Planning/Rwanda, Ministry of Health/Rwanda, ICF International Rwanda Demographic and Health Survey 2014–15. [Internet] Kigali, Rwanda: National Institute of Statistics of Rwanda, Ministry of Finance and Economic Planning/Rwanda, Ministry of Health/Rwanda, and ICF International; 2016; [cited August 3, 2020]. Available from http://dhsprogram.com/pubs/pdf/FR316/FR316.pdf

[26] National Institute of Statistics of Rwanda (NISR), Ministry of Health (MOH)/Rwanda, ICF International Rwanda Demographic and Health Survey 2010. [Internet] Calverton (MD): NISR/Rwanda, MOH/Rwanda, and ICF International; 2012; [cited August 3, 2020]. Available from: http://dhsprogram.com/pubs/pdf/FR259/FR259.pdf.

[27] Central Statistical Agency (CSA)/Ethiopia, ICF Ethiopia Demographic and Health Survey 2016. [Internet] Addis Ababa, Ethiopia: CSA and ICF; 2017; [cited August 3, 2020]. Available from: http://dhsprogram.com/pubs/pdf/FR328/FR328.pdf.

[28] Central Statistical Agency/Ethiopia, ICF International Ethiopia Demographic and Health Survey 2011. [Internet] Addis Ababa, Ethiopia: Central Statistical Agency/Ethiopia and ICF International; 2012; [cited August 3, 2020]. Available from: http://dhsprogram.com/pubs/pdf/FR255/FR255.pdf

[29] De Onis M, Onyango A, Borghi E, Siyam A, Blössner M, Lutter C Worldwide implementation of the WHO child growth standards. Public Health Nutr. 2012;15(9):1603–10. 22717390 10.1017/S136898001200105X

